# Who Should Pay for Interdependent Risk? Policy Implications for Security Interdependence Among Airports

**DOI:** 10.1111/risa.13454

**Published:** 2020-02-22

**Authors:** Gabriel Kuper, Fabio Massacci, Woohyun Shim, Julian Williams

**Affiliations:** ^1^ University of Trento Italy; ^2^ Korean Institute for Public Administration Seoul South Korea; ^3^ Durham University Durham United Kingdom

**Keywords:** Airports, cybersecurity, game theory, interdependent risk, security

## Abstract

We study interdependent risks in security, and shed light on the economic and policy implications of increasing security interdependence in presence of reactive attackers. We investigate the impact of potential public policy arrangements on the security of a group of interdependent organizations, namely, airports. Focusing on security expenditures and costs to society, as assessed by a social planner, to individual airports and to attackers, we first develop a game‐theoretic framework, and derive explicit Nash equilibrium and socially optimal solutions in the airports network. We then conduct numerical experiments mirroring real‐world cyber scenarios, to assess how a change in interdependence impact the airports' security expenditures, the overall expected costs to society, and the fairness of security financing. Our study provides insights on the economic and policy implications for the United States, Europe, and Asia.

## 1 INTRODUCTION

Recent initiatives introduced by the Single European Sky ATM Research Programme (SESAR) and the U.S. Next Generation Air Transportation System (NextGen) have mandated an increased use of integrated information and communications technology (ICT) in Air Traffic Management. An inherent requirement of these initiatives is a tightly integrated and interlinked airport information network resulting in an increased security interdependence among airports. A clear consequence from the U.S. Federal Aviation Administration (FAA) Administrator M. Huerta's own words in 2011 is that “*With that evolution [NextGen] the cyber security risks will increase*.”

Cyber‐physical network dependency, for which aviation is a prime archetype, is a key problem, both literally in the sense of information networks and more generically in the sense of interconnected digital and physical services (Ganin et al., 2020; Gisladottir, Ganin, Keisler, Kepner, & Linkov, 2017).

This article introduces a simple, but rich, model of interconnected risks. The model has an explicit and an implicit mechanism which generate interdependencies between network nodes (in this case, individual airports). The explicit mechanism assumes positive externalities between nodes (individual airports) that are generated by the degree of interconnectedness. Security expenditures of connected nodes can decrease the probability of attack of a target along the model of the Heal and Kunreuther analysis of interconnected airports (Heal & Kunreuther, 2007). For example, interconnectedness can be proportional to routed traffic connections so that interdependency is empirically and monotonically related to the underlying degree of activity connecting the airports, based around a measure of traffic, a route we pursue in this article for a simulation of our results. A second, implicit mechanism is due to the activities of attackers that is determined by the underlying attractiveness of airports as targets. The first type of dependency has been suggested, with varying degrees of complexity, in several prior studies (Hausken & He, 2016; Paté‐Cornell, Kuypers, Smith, & Keller, 2018; Shafieezadeh, Cha, & Ellingwood, 2015). The second type, and the interaction with attacking intensity operating across the network permits the analysis of the effect of different policies on security. In this instance, our interest is in the fairness of the allocation of funding through passenger taxation schemes.

From the viewpoint of a reduction in the possibility of an adverse event air security can reasonably be modeled as superadditive in nature, by construction. That is, the joint contribution to security of individual actions is greater than the simple sum of the individual parts. Furthermore, this superadditive security risk reduction is transversal, that is, the endowment of security is distributed across individual airports. This combination of effects is often referred to as a positive network‐externality. Several prior studies have either directly or tangentially addressed this type of externality in the security context (Chopra & Khanna, 2015; Haphuriwat & Bier, 2011; Insua, Cano, Pellot, & Ortega, 2016; Zhang, Ramirez‐Marquez, & Wang, 2015).

In the airport domain, cyber‐threats are not often separate from physical ones.4The Association of Airport Directors (Airport Council International, 2014) have broadly classified cyber‐threats into three groups. The first one concerns subvertible systems, such as operationally critical networks, baggage systems, and web pages. The second group includes directly theft and fraud resulting in direct financial losses for airlines, onsite vendors, passengers, employees, and contractors. The final group includes all attacks related to terrorism. Risks arise from the interaction of vulnerability, threat‐actor (we use the catch‐all phrase “attacker”) and individual plus joint threat mitigation efforts. Cyber attacks in conjunction with physical attacks may be used to increase potency or simply to be the core focus of an effort to exploit cyber to physical effects (Hausken, 2019; Zhuang, Bier, & Gupta, 2007), for instance by malicious attacks on Supervisory Control and Data Acquisition systems (SCADA) or other critical equipment; or embarrass commercial entities and act as a conduit for a political message (Hausken, 2017; Nganje, Bier, Han, & Zack, 2008; Wu, Tang, & Wu, 2015).

If security threats increase, more security countermeasures need to be deployed to mitigate risk. An important policy question is how these mitigation measures are to be financed. At present, average security costs by airport account for approximately 35% of overall airport operating costs, and are financed using a variety of mechanisms. For example, in the United States, security costs are partly funded through direct taxes (a flat rate tax of $5.6 per passenger/flight segment), and mostly from the Federal Government (Gulliver, 2014). In Europe, there is a larger variety of models (Falconer, 2008; Irish Aviation Authority & Aviasolutions, 2004). Some countries (e.g., Finland, Germany, Italy, and Switzerland) follow a centralized financing model (states collect taxes centrally and redistribute them to airports for funding security costs), other countries (such as Belgium, Denmark, France, and the United Kingdom) follow a decentralized model, where security is the responsibility of the airport; under the supervision of a central authority, airports directly pay for security through charges imposed on passengers. Yet, whether collected from the airport or from the state, the final outcome is a flat rate levied on a per‐passenger basis (Irish Aviation Authority & Aviasolutions, 2004) ranging from €5 to €7. This levy, as in the U.S. model, is, unfortunately, hardly enough to cover the costs: *“In 12 of the 13 [European] States with operating deficits […], the airports fund the major proportion of the deficit”* (Irish Aviation Authority & Aviasolutions, 2004, p. 48).

One of the notable results is that even though mandated security expenditures minimize the total expected costs to society from an attack under the current security financing scheme, the distribution of security expenditures for airports of different natures would be unfair. This study also shows that the new ICT‐based operational initiatives that affect interdependence among airports might be beneficial to small airports, as they can reduce an unfair burden of security expenditures.

## 2 A BRIEF REVIEW OF THE LITERATURE

Conceptually, our baseline assumptions on the structure of dependency follow the canonical definitions put forward in the survey by  Hausken and Levitin (2012): *“Systems where an impact on one element gets transferred further to one or several other elements due to linkages”(Hausken & Levitin*, 2012, *p. 356). Among the several type of systems described in the survey (single elements, network, etc.), the interdependent systems subsume the network and the multiple elements as it makes explicit the effects of linkages*.

Methodologically, there are a number of modeling approaches that can be deployed to achieve this conceptual framing. Prior research has focused on three main approaches: first, agent‐based simulation (ABS) models that impose a detailed structure on security interactions, but often rely on quite *ad hoc* statistical premises; second, input–output (IO) models, usually entailing simpler discrete event simulations than full agent‐based models that focus on key relationships between objects within the scope of the simulation; finally, partial equilibrium models where an individual agents' actions are modeled in response to statistical summaries of the rest of the system. We shall look at the pros and cons of each technique, before explaining how our framework sits within the current set of canonical models.

The objectives of ABS models are mainly to investigate large‐scale complex interactions, and address issues caused by interdependence in the network. ABS models focus on investigating the decision processes and action strategies of various agents in interdependent infrastructure systems. For example, North (2001), Macal and North (2002), and Veselka et al. (2002) study behavior, strategies and decisions of interdependent infrastructure agents, and model interactions among them. In each case, an ABS model is used to demonstrate how interdependent infrastructures respond to changes in market conditions and disruptions. Further work in Barton, Edison, Schoenwald, Cox, and Reinert (2007) and Brown, Beyeler, and Barton (2004) also develop ABS tools that explore how interdependence impacts network and market performance in the case of large‐scale disruption or policy changes on interdependent infrastructure networks, and how individual agents react to these events.

While ABS models capture system structure quite accurately, their very complexity poses problems. The stochastic properties of individual components often have to be specified through informed guesses on the capabilities of attackers and defenders to change the outcome likelihood of success or failure of an attack. Dependency is often mechanically imposed and the transfer of information can be significantly affected by modeling decisions driven by intuition rather than by data. To overcome some of these issues, several approaches inherit some of the structure of ABS models, but impose certain simplifying structures to permit easier parameterization from experimental data. For instance, Haimes and Jiang (2001) introduced Leontief‐based IO models to analyze the impact of a disruption on interdependent infrastructure systems. Here, the interrelations are linear, and hence the coefficients of the model are those that determine the input and output vector, subject to certain plausible structural restrictions. Further models proposed by Jiang and Haimes (2004),  Santos and Haimes (2004), Santos (2006), and Leung, Haimes, and Santos (2007) increase the degree of complexity within the model (e.g., by increasing the size of input and output vectors), to reveal more complex interactions. However, due to the limitations of traditional Leontief‐based models (e.g., linearity, lack of behavioral content, and lack of consideration of network structure), more recent studies including Zhang and Peeta (2011, 2014) and Resurreccion and Santos (2013) have proposed models incorporating more features such as multiperiod dynamics and nonlinear interactions.

Another area related to our study is the work in security economics. While many of the studies in this field also take into account a perspective of interdependence, they develop a separate spectrum of discussion on the interdependence in the security context. Specifically, these studies explore not only interdependence among economic actors, but also the interaction between the economic actors and adversaries attacking the systems. For example, Florêncio and Herley (2013), Cremonini and Nizovtsev (2009), Fultz and Grossklags (2009), Ioannidis, Pym, and Williams (2013) study the interactions between attackers and defenders in an interdependent security setting. Zhuang et al. (2007) and more recently Hausken (2017) have studied interdependence in the framework of supply chains including transportation  along the lines of McLay, Jacobson, and Kobza (2008). See, in particular, the work of Hausken (2017) for a comprehensive discussion on the limitations of the previous literature in the treatment of interdependence. A key observation is that the previous literature has often assumed that defenses and attacks against one target are also effective against another target, depending on an interdependence parameter. In other words, if a target is attacked, the other ones also suffer from it. Modern approaches, including our own article, conceptualized interdependence in such a way that if one target fails, other targets fail with a certain probability controlled by interdependence parameters linking each pair of targets. Those parameters might, in their turn, depend on the other targets' security expenditures. However, some modeling assumptions are still appropriate and we also use them in this article. For example, Zhuang et al. (2007) represent interdependence as the probability that an attack on agent *i* infects an agent *j* but assume a constant interdependence coefficient for technical aspects in their simulation. We complement it with the amount of traffic among the interconnected airports so that it can vary for different pairs of *i* and *j* (see further in Section 6).

In these cases, specific agents are modeled, or grouped together, such that their actions are considered to be identical in expectations. For example, deriving attacker production functions when the actions and opportunity sets of targets is presumed to be a simple stochastic process. Or vice versa, viewing attackers as random actors with no general strategic goals and modeling defensive investment with and without some form of coordinating regulation (see the cited survey Hausken & Levitin, 2012, for a comparison of different classification of attacks and defense measures). Indeed, tractable models of security investment are often preferred for practical insight. For example, Gillen and Morrison (2015) use a simple discounted cash‐flow approach to establish a cost‐benefit criteria for managing terrorism risk (primarily) in air transport.

## 3 REGULATION, FINANCING, AND OPERATION OF AIRPORT SECURITY

In the United States, air transport security is mostly regulated at the federal level (by the Transport Security Administration, TSA) with some ground installation regulation occurring at the state level. In contrast, within the European Union (Irish Aviation Authority & Aviasolutions, 2004), European Economic Area and the European Free Trade Association, security is primarily a member action with coordination of standards set by the European Commission.5Regulation (EC) No. 2320/2002) and Regulation (EC) No. 300/2008 setting the harmonization of standards in civil aviation security were further advanced by revising and elaborating. One of the main objectives of the EU approach to regulation has been to make aviation security regulation more flexible and up‐to‐date against innovation in attacker technologies. An example of an explicit physical interdependence embedded in regulation is the concept of “one‐stop security” which sets out rescreening procedures for transfer passengers arriving from non‐EU countries (Falconer, 2008). Table I provides an example of the typical security requirements from current regulations.

**Table I:** Examples of “Traditional” Security Measures in Airports from Graham et al. (2013, table 5.4)

Protection Measure
1. Badge regime and reliability check on badge applicants.
2. Checks on access to restricted areas and video supervision.
3. Checks on passengers and hand baggage.
4. Baggage reconciliation and checks on hold baggage.
5. Checks on cargo/airmail.
6. Armed protection land‐side and airside.
7. Protection of parked aircraft.

Asia has a diverse regulatory approach. For example, airports in Australia (InterVISTAS Inc., 2018; Tourism & Transport Forum, 2015), South Korea (Han Young Yoon, & So, 2011), and Singapore (IATA, 2005, 2009; Kandiah, 2004) tend to use the privately operated and government regulated model. In contrast, many airports in China (Kandiah, 2004; LeighFisher Ltd., 2013), Hong Kong (IATA, 2005), and India (Kandiah, 2004; Singh, Dalei, & Raju, 2015) are still owned by the government and depend on the centralized model. See also Gillen and Morrison for a comprehensive survey of severa countries (Gillen & Morrison, 2015).

From the attacker's perspective, there is also a coordinated set of risk assessments that are typically published for European airports by Eurocontrol, see the “Eurocontrol‐manual” (Eurocontrol, 2010). In Table II, we have extracted a snippet of the likelihood classifications for successful attacks against airports and the required controls to reduce exposures. Similar tables are used in SESAR's work package (WP) 16, that manages the risk assessment for SESAR's operational concepts.

**Table II:** Likelihood of a Successful Attack (From Eurocontrol ATM Risk Toolkit)

Likelihood	Physical	People	Electronic
High	Physical access possible	No control or prerequisite engineering knowledge	Normal function or known vulnerability
Medium	Physical barriers in depth	Access control, staff checking & training	Well isolated & access controlled
Low	Protection, inspection & audit	Include separation polices & audit	Segregated networks and regular monitoring

ACI Europe (the airports association) argues that governments should fund civil aviation security centrally, since terrorists commonly target states rather than a specific airport, which are selected as a function of profile and likelihood of success.

Furthermore, there is an incentive problem whereby competition and asymmetric passenger number growth might distort individual airport security investment, see, for instance, ACI Europe (2003, 2009, 2010) for survey evidence on these effects. Furthermore, according to commissioned research from Aviasolutions for the Irish Aviation Authority (Irish Aviation Authority & Aviasolutions, 2004), there have been inconsistent security funding mechanisms across various European countries. Table III outlines the heterogeneity in state security funding approaches. While the centralized model is a commonly used system for security financing, many countries employ a diverse set of funding mechanisms.

**Table III:** Structure of Airport Security Provisions

Funding and Provision Model	Centralized Model	Decentralized Model
Provision of security activities	Austria, Finland, Germany, Iceland, Italy, Luxembourg, Norway, Portugal, Spain, Sweden, Switzerland, USA, China, India	Belgium, Denmark, France, Greece, Ireland, UK, South Korea, Australia, Singapore
Countries charging state security taxes	Austria, Germany, Iceland, Italy, Netherlands, Portugal, Spain, USA, China	Belgium, France
Countries charging airport security charges	Luxembourg, Sweden, Switzerland, Germany, Netherlands, India	Belgium, France, Greece, Iceland, UK, South Korea, Australia, Singapore

Such variety of funding models is coupled with a wide variety of airport types. In interview with stakeholders, airport types are commonly distinguished by their sizes. Large airports are assumed to be airports working as hubs for medium‐small airports, and medium‐small airports are considered to be spoke airports with relatively low traffic volume. The difference in scale among them is illustrated in Table IV.

**Table IV:** Traffic Information on Sample Airports

	Traffic Volume			Passengers/Day Coming from		
Airport	Pass/Year	Flights/Day	Pass/Day	Large	Medium	Small
Large (Munich, DE)	37.7M	680	101.370	18.182	48.205	34.983
Medium (Verona, IT)	2.7M	222	7.397	3.226	1.467	2.704
Small (Ancona, IT)	0.5M	20	1.479	565	652	262

*Note*: Munich is the second hub of Lufthansa in Germany, the 7th European Airport and 27th worldwide; Verona is a “feeder airport” for other national carriers (e.g., Lufthansa to Munich, Alitalia to Rome, etc.) and some low‐cost airlines; Ancona's airport is only served by Lufthansa, the national carrier Alitalia, and three low‐cost airlines (e.g., Ryanair).

## 4 MODEL STRUCTURE AND ASSUMPTIONS

Our model considers strategic interactions between three classes of actors: multiple attackers, airports, and a risk‐neutral social planner regulator for all airports. Following Ioannidis et al. (2013) and the random attack model of  Hausken and Levitin (2012), we assume that targets are strategic and attackers are reactive. The former follows a Nash Equilibrium and the latter a Cournot Equilibrium, and all players move simultaneously.

### 4.1 General Assumptions about Targets and Attackers

Table V presents the critical model variables and parameters for individual agents within the model. Network models with equilibrium decision making have been quite extensively studied before, see Galeotti, Goyal, Jackson, Vega‐Redondo, and Yariv (2010) and Jackson and Zenou (2015) for surveys. While our game has several unique features in terms of the structure of the various interdependencies, the basic decision making per node follows a standard concave game. Risk reduction is log‐linear and realized costs of implementation are linear in investment. This ensures that for a series of relatively mild constraints on parameters, targets have well‐defined optimal security investment choices. Our choice of functional form is driven by the need to have a tractable equilibrium solution which we will then exploit in our numerical simulations to establish the public policy optimum levels of taxation and risk transfer.

**Table V:** Description of Model Parameters and Decision Variables

*Defender/Target*		
*N*	Number of airports	
$x_{i}$	Airport *i*'s security investment.	Endogenous Decision Variable for the *i*th airport.
$\alpha_{i}$	Airport *i*'s marginal risk reduction.	Parameter dependent on defensive technology.
$A_{i}$	Airport *i*'s zero investment risk.	Parameter dependent on technology.
$L_{i}$	Airport *i*'s assets at risk.	Environmental parameter.

*Policy maker*		
$v_{i}$	Social planner's weight for airport *i*.	In a pure utilitarian model, this is an environmental parameter.
$\delta_{\mathrm{ij}}$	Degree of security dependency of airport *i* on airport *j*.	Parameter dependent on technology and connectivity.

*Attacker*		
$N_{A}$	Variable number of attackers	
η	Attacker intensity.	Variable derived from $N_{A}$ decisions to attack against *N* airports.
β	Decay factor in repeated trials.	Parameter dependent on attacking technology.
$\rho_{i}$	Reward/cost ratio for an attack, R/C.	Parameter based on the attackers subjective view of cost and reward given a successful attack on the *i*th target.

Let *i* and *j* index airports such that i≠j∈{1,…,N} are the indices of each airport. The security expenditure made by airport *i* is represented by $x_{i}$ and the vector of security expenditures $\left(x_{1},\cdots ,x_{N}\right)$ by **x**. We assume that the defender and the social planner are optimizers, and therefore that the strategic interaction follows a Nash equilibrium under risk neutrality. The security investments of the risk‐averse players will be obviously higher than the risk‐neutral player and therefore the effects captured in this article will be amplified (see Massacci, Swierzbinski, & Williams, 2017 for a discussion of what happens in the case of cyberinsurance). However, risk neutrality simplifies the mathematical treatment and allows us to focus on the study of interdependence rather than the difference between risk aversion and risk neutrality.

From our cybersecurity perspective, attackers are assumed to be reactive and identical in nature: they have the same characteristics for launching an attack on the cyber‐infrastructure of airports. From the statistical view of the target, airports are attacked by $N_{A}$ attackers, where the likelihood of an attacker being matched to any given airport is identical for all attackers and hence 1/N. If we consider that the number of potential attackers is arbitrarily high, the number $N_{A}$ of actual attackers, hereafter expressed as the number $N_{A}$ of attackers, is endogenously determined by a potential attacker selecting himself into attacking or not attacking. Hence, we can consider a single decision variable, the average number of attacks, denoted as η, on *N* airports as given by the ratio $\eta =N_{A}/N$, which we refer to as the attacker intensity. An alternative view is to assume a fixed number of attackers and that each attacker attacks with a certain endogenously determined intensity η.

Since all potential attackers are assumed to be identical, they have the same cost, *C*, for launching an attack. This refers to software attacks, not attacks on the physical infrastructure. Most common mechanisms of attack are, by construction, mostly indiscriminate (Bilge & Dumitras, 2012). Hence our focus is toward *untargeted attacks* against the organization, typically supported by automatic tools responsible for the vast majority of attacks in the wild  (Bilge & Dumitras, 2012). Targeted attacks are rare events (“Black Swans”) (Nayak, Marino, Efstathopoulos, & Dumitraş, 2014), for which the lack of foresight understanding of the event's causes and dynamics can be better captured by “uncertainty” as opposed to “probability” (Brown & Cox, 2011; Flage & Aven, 2015). The only known attack to an airport (Bristol, see https://www.bbc.com/news/uk‐england‐bristol‐45539841) was indeed caused by a generic ransomware. This may include the cost of setting the cyber‐infrastructure, the cost of incarceration if caught, and the opportunity cost of the lost return from pursuing alternative options (e.g., an attack on the power grid) (Ioannidis et al., 2013).

While we assume identical attackers in terms of means, their expected reward from a successful attack on different airports might differ. This is due to the fact that attackers might be able to achieve a far higher reward by successfully striking a large airport than a small airport. We therefore assume that the expected reward obtained from a successful attack depends on the airport attacked, and use $R_{i}$ as the reward per attack against airport *i* when one or more of these attacks turns out to be successful. We do not make any assumption on the unit in which revenues from successful attacks are measured. They could be kudos in hackers fora (Ooi, Kim, Wang, & Hui, 2012) or revenues from trading victim's assets in black markets (Allodi, Corradin, & Massacci, 2015; Grier et al., 2012) or number of fatalities (Sandler & Lapan, 1988).

We define $\rho_{i}=R_{i}/C$ as the reward/cost ratio against airport *i*. While $\rho_{i}$ inherits its heterogeneity from $R_{i}$, we shall show that from the attacker assumptions the overall risk associated with the attacker decision making will be determined, mostly, by the average value and the structure of the network. A key issue is to actually assess it from empirical data. For example, Ooi et al. (2012) do not report the actual effort required by hackers to deface websites. However, later in the simulation we only need to assume that the cost‐to‐reward ration is reasonably high (a factor of 2 or 10) as suggested by the literature.

We use $\sigma_{i}$ to represent the probability that one or more attacks mounted against airport *i* are successful. Consistent with the previous literature (Ioannidis et al., 2013), this probability $\sigma_{i}$ is conditional on the strategic decisions of attackers and airports. Specifically, we let $\sigma_{i}=\sigma_{i}\left(x,\eta \right)$, implying that $\sigma_{i}$ depends on **x** and η. We assume that $\sigma_{i}$ is considered to have the following properties for all i≠j. Assumptions 1–3 are discussed in Gordon and Loeb (2002) and Ioannidis et al. (2013) and 4 and 5 are natural extensions of these.

Assumption 1 An increase in the security expenditure of a target decreases the probability of a successful attack ($\partial \sigma_{i}/\partial x_{i}<0$).

Assumption 2 There are decreasing marginal returns to security expenditure ($\partial^{2}\sigma_{i}/\partial x_{i}^{2}>0$).

Assumption 3 There is a potential benefit from another airport's security expenditure on the target, hence a direct mechanism of positive externalities of security expenditure ($\partial \sigma_{i}/\partial x_{j}\leq 0$).

Assumption 4 Marginal effectiveness of positive externalities decreases as an airport increases its own security expenditure ($\partial^{2}\sigma_{i}/\partial x_{i}\partial x_{j}>0$).

Assumption 5 An increase in the average number of attacks made against a target increases the probability of a successful attack ($\partial \sigma_{i}/\partial \eta >0$).

Assumption 6 Attackers who have chosen to attack an airport have an equal probability of being matched to any given target (attacker target matching is at maximum entropy). An attack on any given airport is an independent experiment from any other attacks.

### 4.2 Introducing Interdependence among Targets

The security dependency structure is in two parts. First, through a direct channel, where investment has a direct cross product in reducing the probability of successful attacks when attacker intensity is fixed. This is similar to the average dependency structure in Kunreuther and Heal (2003) and Heal and Kunreuther (2003) although we allow for heterogeneous codependency across the network of airports, which is presumed to be exponential affine in structure. To do so, we define the probability explicitly by selecting some functional forms which satisfy our general assumptions (Gordon & Loeb, 2002) (Assumptions 1–6) and have been also explored in similar forms in the literature (Hausken & Levitin, 2012). For example, see Gordon and Loeb (2002), Kunreuther and Heal (2003), or more recently Hausken (2019).

(1) $$\sigma_{\mathrm{base},i}\left(\eta \right)=A_{i}\frac{\eta }{\beta +\eta },$$

(2) $$\sigma_{i}\left(x,\eta \right)=\sigma_{\mathrm{base}\;i}\left(\eta \right)\exp\left(-\alpha_{i}\left(x_{i}+\sum_{j\neq i}\delta_{\mathrm{ij}}x_{j}\right)\right).$$

$\sigma_{\mathrm{base}\;i}\left(\eta \right)$ indicates the baseline probability of a successful attack when there is no security investment and the number of attackers is fixed exogenously at η. $A_{i}$ is the probability that an attack on airport *i* is successful in the absence of any cybersecurity expenditure by the airport or any spillover in security from the network effect. β represents a decay factor6As $\eta =N_{A}/N$ then $\eta /\left(\beta +\eta \right)=N_{A}/\left(\beta \cdot N+N_{A}\right)$. For the simulation, we will assume β=1/N, i.e., almost all attacks will be successful if no countermeasure is taken. as not all independent attacks will be successful (see also the contest parameter (Hausken, 2019)).

From Equation (2), $\alpha_{i}$ is airport *i*'s marginal reduction in risk from additional security expenditure. $\delta_{\mathrm{ij}}$ is the transmission factor that determines how a marginal effect of a change in investment by airport *i* transfer to airport *j* and hence determines the degree of security interdependency. The security level of one airport therefore combines outputs of security efforts of the other airports as well as the airport

Here, we follow Hausken (2019) so that interdependence is conceptually modeled as a conditional probability: a target *i* may fail when target *j* fails. In our scenario, $\delta_{\mathrm{ij}}\geq 0$ means that an increase in the security expenditures $x_{j}$ have a positive impact on the security of agent *i* thus leading to a lower chances of success for an attack to hit agent *i*. In the case of airports, a suitable proxy for the level of interdependency is the passenger traffic between them. We discuss this issue in more detail in Section 6.

When $\delta_{\mathrm{ij}}=1$, airports *i*'s one extra unit of security expenditure affects airport *i* to the same degree as one unit of its own expenditure; when $\delta_{\mathrm{ij}}=0$, there is no security interdependence.

As attackers are reactive they will attack if there is any chance of making a “profit” (in their unit of measure, see previous discussion), thus eventually ending in a Cournot Equilibrium. The expected payoff, $\Pi_{i,A}$, from mounting an attack on airport *i* is given by

(3) $$\Pi_{i,A}=\sigma_{i}\left(x,\eta \right)R_{i}-C.$$

We consider that the matching of an attack to an airport is at maximum entropy, hence the probability of any attacker who has chosen to attack any airport is 1/N, as such the expected payoff for any given attack, indexed by *a*, is $E\left[\Pi_{a}\right]=1/N\sum_{i=1}^{N}\Pi_{i,A}$. The equilibrium total cost $CN_{A}$ versus expected reward $\sum_{i=1}^{N}\sigma_{i}\left(x,\eta \right)R_{i}$ when each η, should satisfy:

(4) $$\sum_{i=1}^{N}\sigma_{i}\left(x,\eta \right)R_{i}=CN_{A}.$$

While the left‐hand side of Equation (4) shows an attacker's aggregate reward from an attack made against the whole population of target airports, the right‐hand side of the equation is an aggregate cost for all attackers to engage in attacks.

This equation ensures that more attacks will be launched as long as the expected reward from an attack (left side of the equation) exceeds the cost of the attack (right side of the equation) (Ioannidis et al., 2013). Rewriting Equation (4) and dividing it by *CN* determines the equilibrium attacking intensity where we replace $R_{i}/C$ with $\rho_{i}$ and $N_{A}/N$ with η (see Table V):

(5) $$\frac{1}{N}\sum_{i=1}^{N}\sigma_{i}\left(x,\eta \right)\rho_{i}=\eta .$$

It should be noted that, in equilibrium, the average number η of attacks per target satisfying Equation (5) depends in general on the vector x of security expenditures by the *N* airports.

We let $L_{i}$ represent the expected loss suffered by airport *i* when one or more successful attacks on the airport occur. Without loss of generality, we suppose that $L_{i}$ does not depend on the total number of successful attacks but only on whether there is a single successful attack. This is appropriate for airport security, as a severe incident in an airport is routinely followed by detailed security action and the vulnerable point that caused the incident is removed. Therefore, airport *i* will select its level of security expenditure $x_{i}$ by minimizing the expected cost

(6) $$V_{i}^{*}=\min_{x_{i}}\left[\sigma_{i}\left(x,\eta \right)L_{i}+x_{i}\right].$$

### 4.3 Introducing a Social Planner

Assumption 7 We assume that the airport regulator is a Stackelberg policy maker who seeks to minimize the total expected cost, calculated by the weighted average of the individual expected costs (expected loss from attacks plus investments) of each airport. The policy is able to individually set minimum investment levels in security for each airports.

To take into account the regulator in the analysis, we assume that the regulator sets the vector of the levels of security expenditures for all airports, $x^{\prime }$, to minimize the weighted average of the airports' expected costs, $\sum_{i=1}^{N}v_{i}\left(\sigma_{i}L_{i}^{\prime }+x_{i}^{\prime }\right)$, where $v_{i}$ are positive weights indicating how much importance the regulator places on airport *i* and $L_{i}^{\prime }$ is the loss appreciated by the policy maker (e.g., fatalities or reputation). The vector $\left(x_{1}^{\prime },x_{2}^{\prime },\cdots ,x_{N}^{\prime }\right)$ is the social planer or regulator's strategic choice variable.

Normally, regulators treats all airports equally, or at least claim to do so. For example, interviews with key stakeholders showed that this is a common practice of the European Commission (De Gramatica, Massacci, Shim, Tedeschi, & Williams, 2015). So one could also assign $v_{i}=1$.

It is however possible to treat the general case of $v_{i}\neq 1$ by absorbing weights into the losses and investment by setting $L_{i}=v_{i}L_{i}^{\prime }$ and $x_{i}=v_{i}x_{i}^{\prime }$. Then one needs only to replace $x_{i}$ with $x_{i}^{\prime }/v_{i}$ in the derivations and in the propositions to obtain the weighted results.

For a social planner regulator, the objective function reduces to the following expected cost minimization problem:

(7) $$\min_{x_{i}}\sum_{i=1}^{N}\left(\sigma_{i}L_{i}+x_{i}\right),$$

where each airport is effectively weighted by the size of the loss incurred in the event of a successful attack. As already mentioned, we restrict our analysis to the risk‐neutral case. See Massacci et al. (2017) for how security investments are amplified when risk aversion is considered in the case of cyberinsurance.

## 5 INTERDEPENDENT SECURITY WITH STRATEGIC ATTACKERS

We first consider the base case where there is no security interdependence, hence $\delta_{\mathrm{ij}}=0$ ∀i,j∈{1,…,N}. Simultaneously, the two sets of player types choose their equilibrium expenditure on security investment following Equation (6) and the number of attackers $N_{A}$ is determined by the expected cost versus expected reward tradeoff from Equation (5). The simultaneous Nash equilibrium is the combination of optimal security investments $x_{i}^{*}$ and attacker intensities $\eta^{*}$ that jointly satisfy the objectives in Equations (6) and (5).

### 5.1 Equilibrium in Absence of Direct Interdependence

Denoting the equilibrium level of attacks per target as $\eta^{*}$, the first‐order condition characterizing the optimal security expenditure of airport *i*, $x_{i}^{*}$, can be written as

(8) $$\frac{\partial \sigma_{i}\left(x,\eta^{*}\right)}{\partial x_{i}}L_{i}=-1.$$

The second‐order condition for optimality is satisfied implicitly by our assumptions on decreasing marginal returns for security investments (see Assumptions 2 and 4). By setting $\delta_{\mathrm{ij}}=0$, reorganizing Equation (8) with respect to $x_{i}^{*}$, and solving this and Equation (5) simultaneously, we get the following result.

Proposition 1 (Nash equilibrium with no direct security interdependence) When $\delta_{\mathrm{ij}}=0$, the Nash equilibrium $\left(x^{*},\eta^{*}\right)$ for airports and attackers can be defined for all airports *i* as

(9) $$x_{i}^{*}=\frac{1}{\alpha_{i}}\log\left(\alpha_{i}L_{i}\cdot \sigma_{\mathrm{base},i}\left(\eta^{*}\right)\right),$$

(10) $$\eta^{*}=\frac{1}{N}\sum_{1\leq i\leq N}\frac{\rho_{i}}{\alpha_{i}L_{i}}.$$

Proposition 1 shows that the level of equilibrium security expenditures in airports is mainly determined by the marginal loss reduction from additional security expenditure and the baseline probability of a successful attack. The equilibrium number of attackers depends on the ratios between the reward for an attack on airport *i* and the marginal effect of security expenditure on loss reduction, $\alpha_{i}L_{i}$.

We now consider a case where an airport regulator seeks to identify the optimal regulatory intervention for the airports.

Proposition 2 (Optimal strategies under regulatory intervention with no direct security interdependence) If there is no security interdependence, the regulatory intervention yields the following responses $\left(x^{†},\eta^{†}\right)$ from airports and attackers for the *i*th airport:

(11) $$x_{i}^{†}=x_{i}^{*},$$

(12) $$\eta^{†}=\eta^{*}.$$

From Proposition 2, it follows that without security interdependence, the regulatory intervention yields the same result as the Nash equilibrium and there is no need for regulatory intervention to set minimum security investment levels.

### 5.2 Equilibrium in Presence of Direct Security Interdependence

We now investigate the effects of security interdependence on the strategic behavior of airports and attackers. As security interdependence is taken into account, airports' security expenditures benefit not only themselves, but also the airports connected to them. Thus, we need to consider strategic interactions not only between the choices of attackers and airports, but also between the decisions of different types of airports.

Let Δ be an N×N matrix, such that for each $\Delta_{\mathrm{ij}}$

(13) $$\Delta_{\mathrm{ij}}=\left\{\begin{array}{cc} 0 & i=j,\\ \delta_{\mathrm{ij}} & i\neq j, \end{array}\right.$$

where $\delta_{\mathrm{ij}}$ has been introduced in Section 4 (see Table V). Notice that I+Δ is positive semidefinite and thus (I+Δ)y=x where *I* is the n×n identity matrix implies that y≤x.

Proposition 3 (Nash equilibrium with security interdependence) If there is security interdependence, the Nash equilibrium for the network of airports and its attackers yields the following responses $\left(x^{★★},\eta^{★★}\right)$ from airports and attackers for the *i*th airport:

(14) $$x^{★★}={\left(I+\Delta \right)}^{-1}x^{*},$$

(15) $$\eta^{★★}=\eta^{*}.$$

Proposition 3 indicates that the Nash equilibrium security expenditure for airport *i* depends on the expenditures of other airports but that the number of attackers is not affected. Equations (14) and (15) also imply that for all airports *i* the probability of a successful attack will not change ($\sigma_{i}^{★★}=\sigma_{i}^{*}$).

This phenomenon is consistent with the community equivalent of Kunreuther and Heal (2003) pairwise prisoner's dilemma: the tragedy of the commons. When other people contribute to your security you can keep your current expenditure, and thus improve the overall security level or, most likely, keep your current level of security and lower your costs. The presence of interdependence means that targets can benefit from the expenditures of interconnected targets. However, they focus on their *own* utility (balancing the probability of a loss with the certainty of their own security expenditure), so that *targets will use the benefit gained by interdependence to lower their own expenditure*. As a result, the number of attackers will not change as the overall probability of success remains the same but targets will spend less for security. As someone else will take care of screening, one can avoid double screening and just make sure screened and unscreened do not mix. As we mentioned, this is mathematically apparent from the fact that $\left(I+\Delta \right)x^{★★}=x^{*}$ and therefore $x^{★★}\leq x^{*}$.

Heal and Kunreuther (2007) present a more extreme situation where coalitions of defenders decide to invest and other coalitions may not invest. Such scenarios happen because their model only offers a binary mechanism of choice (invest or not invest) and has no explicit attacker. Hence, there might not be a global equilibrium if the graph is not fully connected. In contrast, not only do we have a continuous mechanism and the choices of the attackers also generate some interdependence (see Massacci et al., 2017, for a discussion of how this happens) but we always have an equilibrium when Δ (Equation (13)) is invertible. This is the case when the eigenvalues of the binary network are nonzero and the graph is connected which is in our practical scenario: you can always fly from anywhere to anywhere else in Europe through some hops. So we do not concern ourselves with coalitions.

Similarly to Proposition 2, optimal strategies of airports and attackers under regulatory intervention with security interdependence can be identified by using Equations (7) and (5) as functions of $x_{i}^{*}$ and $\eta^{*}$. Our solution strategy is to show that when we introduce security interdependence through the matrix Δ, we can express the Nash equilibrium in terms of the case when security is independent with an adjustment for the dependency structure. This is described explicitly in the following proposition.

Proposition 4 (Optimal strategies under regulatory intervention with security interdependence) In the case where there is security interdependence, the regulatory intervention induces the following strategies of airports ($x_{i}^{‡}$) and attackers ($\eta^{‡}$):

(16) $$\sigma^{‡}=\sigma^{*}⊙{\left(I+\Delta \right)}^{-1}1,$$

(17) $$\eta^{‡}=\frac{1}{N}\sum_{i}\frac{\rho_{i}}{\alpha_{i}L_{i}}{\left[{\left(I+\Delta^{T}\right)}^{-1}1\right]}_{i},$$

(18) x‡=(I+Δ)−1x∗−1α⊙log(I+ΔT)−1×1α1⊕σbase,i(η∗)⊘σbase,i(η‡),

where ⊙ and ⊘ are, respectively, the elementwise Hadamard product and division and $\frac{1}{\alpha }$ is shorthand for $1⊘\alpha =[\frac{1}{\alpha_{1}},\ldots \frac{1}{\alpha_{N}}]$.

The regulatory intervention in presence of interdependence has a significant impact on the attacker strategies, as we illustrate in the next subsection. At first, the overall number of attackers per target is lower than with a Nash Equilibrium ($\eta_{i}^{‡}\leq \eta_{i}^{*}$) and the intervention of the regulator has reduced the probability of a successful attack with respect to the attack probability in a Nash equilibrium ($\sigma_{i}^{‡}\leq \sigma_{i}^{*}$). However, it is not necessarily true that the security expenditures have decreased, i.e., it might be the case that $x_{i}^{‡}<x_{i}^{*}$ or $x_{i}^{‡}>x_{i}^{*}$, depending on the level of interdependence.

### 5.3 Analyzing Security Interdependence

To get more insights on how security interdependence affects the strategic decisions of airports and attackers, we compare the results of Sections 5.1 and 5.2. There are two channels for changes in security in expenditure in airport *i* to affect the expected cost in airport *j*. The first is directly via the matrix Δ the first‐order effect. The second is via the externality created by the number of attackers choosing to attack changing as targets adjust their security posture and change the cost–benefit ratio for the attackers.

Imposing structural restrictions on the shape of Δ can yield solutions with more tractable interpretations and analysis. We first compare the difference between the Nash equilibrium security expenditures with and without security interdependence. Since the entries in Δ are all nonnegative, Equation (14) implies that $x_{i}^{*}\geq x_{i}^{★★}$ for all *i*. Formally,

Corollary 1 Under security interdependence, each airport's Nash equilibrium level of security expenditure is less than or equal to the equilibrium level of security expenditure without interdependence.

As such, Corollary 1 indicates that, with increasing security interdependence, airports are likely to underinvest in security, relative to the social optimum under perfect coordination. This result is consistent with the final discussion on the size of a minimum intervention by a regulator for interdependent airports by Heal and Kunreuther (2007) albeit they do *not* have an explicit attacker. It shows the robustness of the conclusion as it comes independently of the details of the actual mathematical formulation (likely because the same broad assumptions are met).

Corollary 2 The probability of a successful attack determined by Nash equilibrium is not affected by security interdependence.

Comparing airport *i*'s equilibrium expected cost with and without security interdependence, denoted as $V_{i}^{★★}$ and $V_{i}^{*}$ respectively, gives us, from Equation (6),

(19) $$V_{i}^{★★}=\sigma_{i}^{**}L_{i}+x_{i}^{★★}\leq \sigma_{i}^{*}L_{i}+x_{i}^{*}=V_{i}^{*}\;\text{for}\;\text{all}\;i.\;$$

This implies that, at the Nash equilibrium, the total expected cost to society with interdependence, $\Sigma_{i}V_{i}^{★★}$, is less than or equal to the total expected cost to society without interdependence $\Sigma_{i}V_{i}^{*}$. Therefore:

Corollary 3 At the Nash equilibrium, an airport's expected cost and the expected cost to society determined by ($x^{★★},\eta^{★★}$) are less than or equal to those determined by ($x^{*},\eta^{*}$).

The regulatory intervention in presence of interdependence has a significant impact on the attacker strategies. Using the same observations on I+Δ, we conclude that $\sigma^{‡}⊘\sigma^{*}\leq 1$, and therefore $\sigma_{i}^{‡}\leq \sigma_{i}^{*}$. The same reasoning applies to $\eta_{i}^{‡}\leq \eta_{i}^{*}$ as it is the sum of the terms of $\eta^{*}$ (Equation (10)) except that each of them is weighted by a number ${\left[{\left(I+\Delta^{T}\right)}^{-1}1\right]}_{i}\leq 1$. The intervention of the regulator has reduced the probability of a successful attack with respect to the attack probability in a Nash equilibrium. However, it is not necessarily true that the security expenditures have decreased, i.e., that $x_{i}^{‡}<x_{i}^{*}$.

We now explore how security interdependence affects the outcomes with regulation. From Proposition 4, Equation (18) can be rewritten as:

(20) $$x_{i}^{‡}=x_{i}^{†}-\frac{\delta_{j}}{\alpha_{i}}x_{j}^{‡}+\frac{1}{\alpha_{i}}\Gamma_{i}\;\text{for}\;i\neq j.$$

At the equilibrium, the number of attacks per target under regulatory intervention $\hat{\eta }$ is also affected by security interdependence and the airport composition (i.e., $\eta^{‡}\neq \eta^{†}$). From Equation (17), we can identify that it is indeterminable whether $\eta^{‡}$ is greater or less than $\eta^{†}$ since security interdependence increases $\eta^{‡}$ by raising 1/(1−Δ). Thus,

Corollary 4 The average number of attacks per target under regulatory intervention with security interdependence is always less than the average number without security interdependence.

Corollary 5 The probability of a successful attack determined by regulatory intervention with security expenditure is less than the probability with no security interdependence.

## 6 NUMERICAL ILLUSTRATIONS

We illustrate numerically the theoretical results and explore the relative magnitudes of the effects for those cases in our policy setting. We first present the calibration of the parameters, and then examine how changes in a policy arrangement affecting security interdependence influence the players' strategies and the overall expected costs for both Nash equilibrium and social optimum cases.

A generalized and comprehensive understanding must take precedence in order to make quantification of the parameters used in the model. Therefore, we first explore the features related to airport security and then provide an example of how the model illustrated in the previous section is parameterized to replicate certain features of European airports. The main motivation of this section is to observe different outcomes of policies where security interdependence is present. The model considers the Nash equilibrium solutions as a *status quo* and calculates the impact of government policy and altering variables in the equilibrium such as the level of interdependence between different types of airports.

### 6.1 Parameter Calibration

For presentational purposes, we divide our analysis into large and small/medium airports, a division which is also used for terrorism studies  (Jacobson, Karnani, & Kobza, 2005; McLay et al., 2008; Virta, Jacobson, & Kobza, 2003) to report costs and impact.

**Fig. 1:**
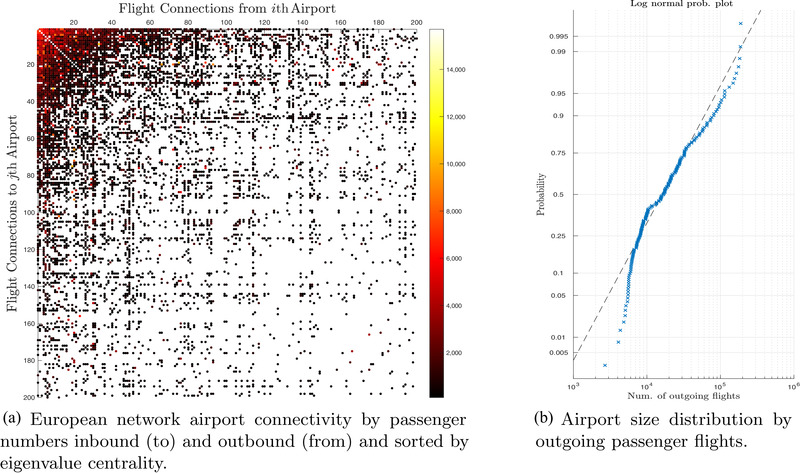
Structure of the European Airport Network by number of passengers.

There is no universal criterion or definition for classifying airport sizes. For example, while the U.S. Department of Transportation uses the total paved runway area to classify airports (Federal Aviation Administration, 2003), the U.S. Congress uses passenger enplanements for classification (U.S. Congress, 1984). Fig. 1 reports the data obtained from Vitali et al. (2011) where 509 European airports are sorted by the number of inbound flights per day. We consider only the airports with a minimum of 5,000 flights per year, which amounts to around a dozen flights per day. For example, Ancona airport in Table IV is included. We also obtain turnover and passenger throughput.

We also derive some of the parameter values from formal and informal interviews with various stakeholders (De Gramatica et al., 2015) to gauge average annual levels of risk and the degree of loss from a successful attack.

We consider as large airports those which have a number of outgoing flights larger than three median absolute deviations away from the overall median. That is: $\mathrm{median}\left(A_{i}\right)>3\cdot \mathrm{median}(|A_{i}-\mathrm{median}\left(A_{i}\right)\left|\right)$. This yields 51 large airports and 135 medium‐small airports in the EU (including the United Kingdom at the time of writing). The distribution of those flights is reported in Fig. 1. It is essentially a log normal distribution. A more refined division would include mega‐hubs and “moderately large” airports. For simplicity, we scale all data (cost, impact, losses, etc.) by dividing all traffic by the median traffic of large airports used as a reference point which amounts to around 60,000 flights. Alternative solutions are possible with a nonlinear scaling.

In addition, we assume that there are on average 100,000 daily passengers for a large airport (see Table IV for an example). The passengers of other airports are scaled according to the respective proportion of outgoing traffic. Leveraging from intelligence information obtained from the U.S. Transportation Security Administration (TSA), some terrorism studies (Jacobson et al., 2005; McLay et al., 2008; Virta et al., 2003) estimate that the cost caused by a security attack (a false clear) in an airport can result in a loss of €1.4B. We use this value as our proxy value for the loss from a successful attack on a large airport. Small/medium airports are scaled similarly by keeping the proportion with outgoing traffic.

Fig. 1(b) illustrates how this assumption yields essentially two groups of airports whose losses are exponentially distributed with a very good fit.

To identify $\alpha_{i}$ from $\sigma_{i}$, recall that $\alpha_{i}$ captures the effectiveness of security investments $x_{i}$. In our scenario, the current security investment is determined by the *security taxes* ${\mathrm{fee}}_{i}$ paid by the outgoing passengers, which we considered proportional to the number of outgoing flights $o_{i}$ per day. From the discussion reported in the various interviews documented in De Gramatica et al. (2015), there is a clear articulation that current airport regulators are attempting to ensure that the number of incidents expected for several years in a row (10 or more) is close to zero. We can assume that the current level of security investments without interdependence is sufficient for the success of attacks to be below the threshold even if we assume that all attacks could be successful $\sigma_{base}=A_{i}$. So if $d_{i}$ is the number of days without incidents we have at least

(21) $$\begin{array}{ccc} \sigma_{i}\cdot o_{i}\leq \frac{1}{d_{i}} & ⟹ & A_{i}e^{-\alpha_{i}\cdot {\mathrm{Passengers}}_{i}\cdot {\mathrm{fee}}_{i}}\cdot o_{i}\leq \frac{1}{d_{i}}\\ & ⟹ & e^{\alpha_{i}\cdot {\mathrm{Passengers}}_{i}\cdot {\mathrm{fee}}_{i}}\geq A_{i}\cdot o_{i}\cdot d_{i}, \end{array}$$

which can be simplified as

(22) $$\alpha_{i}\geq \frac{1}{{\mathrm{Passengers}}_{i}\cdot {\mathrm{fee}}_{i}}\log\left(A_{i}\cdot o_{i}\cdot d_{i}\right).$$

**Fig. 2:**
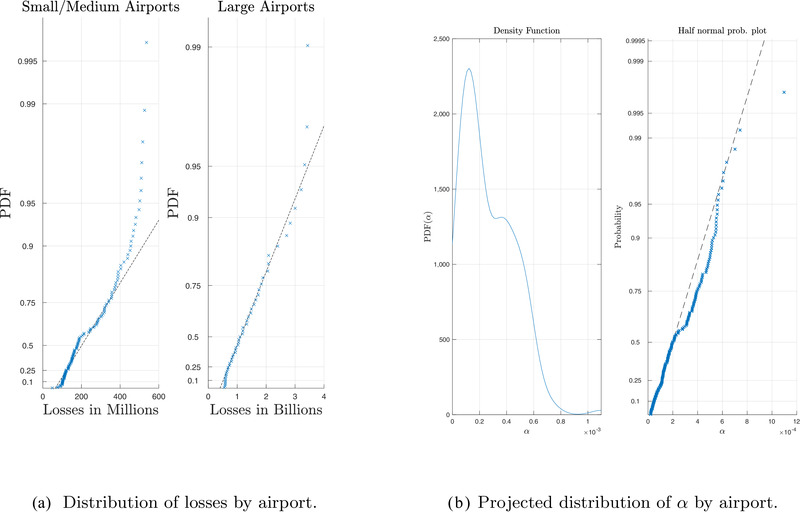
Data used to calibrate the policy simulation.

The parameters $o_{i}$ and ${\mathrm{Passengers}}_{i}$ are derived from the data that we already discussed and, as also mentioned, there is an expectation that $d_{i}\geq 10*365$. From the review of annual reports of various airports (e.g., ACI Europe, 2010; Irish Aviation Authority & Aviasolutions, 2004) and interviews (De Gramatica et al., 2015), we have identified that ${\mathrm{fee}}_{i}$ is between €5 and €7, and hence have used half of the average value (€3) since it must be split between the arriving and the departing airport. The results are presented in Fig. 2(a).

The interdependence coefficient $\delta_{\mathrm{ij}}$ is the contribution of the expenditures of the *j*th airport to the *i*th airport. It is calculated by assuming that it is composed of two factors: a fraction of the traffic volume from the *j*th airport to the *i*th airport with respect to the total traffic of the *j*th airport ($o_{\mathrm{ji}}/o_{j}$), and a security interdependence coefficient. We assume that this interdependence coefficient is constant  (Zhuang et al., 2007) and we show what happens when it varies globally (χ=0.1,0.02,0.001). As for the decay factor of successful attacks, β, we assume that there is a very limited decay and set β=0.001 (see the discussion on the context parameter in Hausken, 2019).

Calibration of the parameter values for attackers is difficult. A point estimate of reward/cost ratio for attacks on type *i* airports, $\rho_{i}$, is adopted from Pym, Williams, and Gheyas (2014), where the reward–cost ratio for cyber attackers is 10. Another estimate from Hausken (2019) claims that the attacker has twice the advantage as the defender ($\rho_{i}=2$).

### 6.2 Results of Numerical Experiments

From the identified parameter values, we can investigate how an ICT‐integrated policy arrangement that changes interdependence affects the players' strategic decisions and the overall performance of the airport network. Our numerical illustrations provides an overview of the intuition of this question. For a specific operational evaluation, one would need expert input on threat intelligence on the value of a particular airport for the attacker and the level of defenses currently in place (see also fig. 2 in Hausken, 2019).

**Fig. 3:**
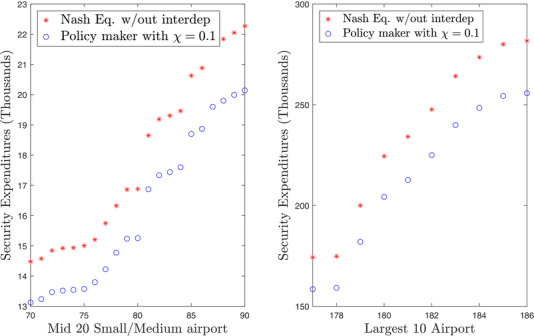
The impact of interdependence for European airports. *Note*: Red dot airports self‐organize under the simultaneous Nash equilibrium when the attackers are the only mechanism for interdependency. Blue dots are airports regulated to globally optimize for interdependence. The latter benefit more in absolute value but almost the same in percentage (range 91% vs. 90–89%) as their expenditures are already much larger.

Fig. 3 illustrates how interdependence can change the security expenditures at Nash Equilibrium. We compare the policy intervention to the Nash equilibrium in absence of interdependence. One can see that both types of airport will benefit from the policy actions albeit the large airports benefit more in absolute value.

An interesting what‐if scenario happens if the policy maker opts for a *simple‐to‐implement policy*: mandate security expenditures in proportion to outgoing passengers numbers. A key question is how far is this policy from the optimal security expenditures. In our scenario, we assumed that Δ is proportional to the number of flights, so such a scenario seems plausible. Consider the gap between the proportional repartition of security expenditures and the optimal level. Consider first the global amount of per‐passenger fees that should be *globally* collected to achieve an optimal allocation, and then see how this value changes in presence of increasing level of interdependencies. For each of these interdependency levels, we also consider the fee that would be collected by large and small/medium airports if they were to individually use their optimal allocated expenditure according to the Nash equilibrium.

**Fig. 4:**
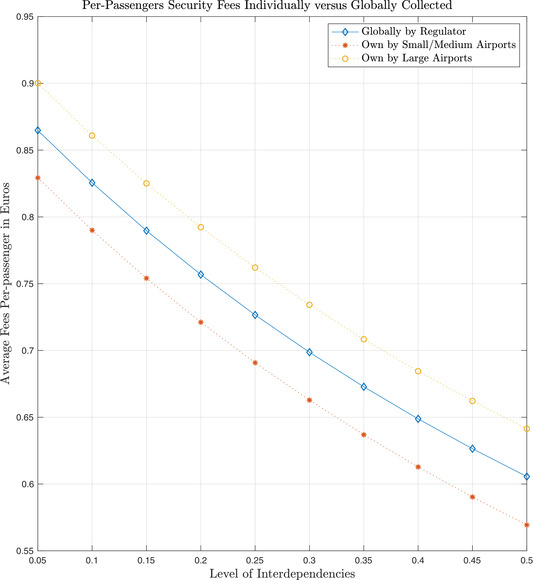
A simulated policy experiment for European airports: Security levy per passenger.

Fig. 4 shows what could happen if the policy maker were to collect all taxes per passengers in a total that was sufficient to cover the overall security costs, but distributed them in proportion to the traffic. What is more important, is that the level of security fee fixed by small airports is always below the one fixed by the regulator for the large airports. For different combination of parameters, we also found out that there is a crossover point after which the regulator asks large airports to pay less and asks small airports to pay disproportionately more.

Finally, we consider what‐if the *increase in interdependence is not uniformly distributed*. The uniform increase that we discussed above can be instantiated for SESAR's integrated IT system SWIM which increases the overall security interdependence in the airport network. The U.S. initiative NextGen has an analogous counterpart.

**Fig. 5:**
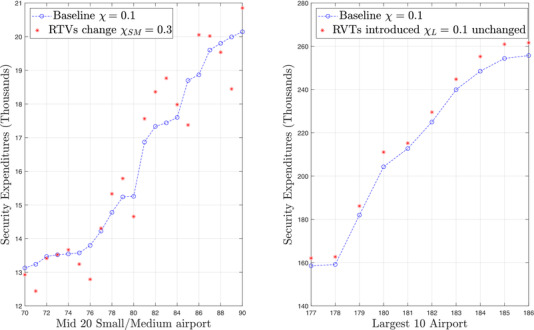
Introducing remove virtual towers: Nonuniform increase in interdependency among European airports. *Note*: Change in security expenditures due to centralizing small/medium airports towers into remote virtual towers. The small/medium airports benefit more of interdependencies. Larger airports are also asked to contribute more as the small airports become also more tightly knit.

A concrete case in which interdependence *increases only in small and medium airports* is the increasing deployment of airport *remote and virtual towers* (RVTs). An RVT replaces a traditional, physical airport control tower by a virtual one in which the view over the window is replaced by a number of sensors which stream their reading to a remotely connected location that manages several airports at once. This cost optimization measure has been in study for several years (SESAR, 2012) and has already started to be piloted and deployed in very small airports (e.g., in Norway and Colorado). This measure which is likely to increase the security interdependence of medium‐small airports (where the RVT will be deployed) over large airports which will still have their own control tower. If the RVT is breached by an attacker, it will make small airports connected to the RVT inoperable. The corresponding simulation in Fig. 5 is particularly interesting because it shows that even if the increase in interdependence only affects a subset of the airports, the others are also affected: almost all large airports have to face an increasing expenditure of several hundred thousand euros. Small airports do not necessarily have to pay less. As is clear from the figures *some* have to pay less but others have to pay more.

In summary, these results imply that technical or policy arrangements which increase security interdependence not only have “functional impacts” (e.g., cost saving) but also a major impact on the security performance and the security financing of the airport network.

## 7 CONCLUSIONS

Understanding network effects and dependencies is a key issue in risk analysis. State‐of‐the‐art results, including this article, are always a tradeoff between the ability to obtain a general solution that is easy to analyze and the choice of a functional form for some aspect of the model. For example, the choice of additive payoffs weighted by probability of success for attackers started from Kunreuther and Heal (2003) and is essentially the same in much of the literature up to 2019 in Hausken (2019) and this article.

The methodological contribution of this study is twofold. First, we outline a tractable network model which enshrines the notion of an expected benefit maximizer seeking to optimally reduce risks through security investment. We then illustrate how the solved form of this model (under both a simultaneous Nash equilibrium and a risk‐neutral social planner regulator), can be used to study policy problems by means of simulation.

A limitation of several interdependency papers, including this one, is that there is so far no generally agreed procedure for measuring and empirically assessing interdependency parameters. For example, this procedure is specified neither in Zhuang et al. (2007) nor in Hausken's papers (Hausken, 2019; Hausken & He, 2016) and in several papers mentioned in Hausken and Fe's survey (Hausken & Levitin, 2012). For simpler models and case studies, e.g., case study three in  Paté‐Cornell et al. (2018), some empirical validation has been proposed, but the process is only sketched. In other cases, the parameter values are drawn from expert opinion (Haphuriwat & Bier, 2011) or the procedure by which the data are provided is not described due to security reasons (McLay et al., 2008). In this article, we have tried to ground the value for our simulation as much as possible on actual data of interconnections from airports, using actual number of interconnecting flights as a reasonable proxy.

When faced with the cost of mitigating risks, those paying need to have some assurance that the costs they face are proportional to the costs of risk burden mitigation that are incurred because of their actions. This basic concept of fair risk sharing underpins a great deal of how a global society operates. The objective of our model is to illustrate the effect of a network dependency structure on both security and the cost of security in an airport setting.

In this article, we use flight data describing the number of connections between airports to capture the degree of interdependency. More granular data could be used to augment this analysis by looking at specific live connections and overlapping systems that might not be captured by the raw passenger data. Indeed, as far as the authors know this is one of the very few *n*‐player interaction games with a closed‐form solution for a general equilibrium that allows different levels of interdependencies between actors. The model automatically adjusts for changes in risk caused by more aggressive and capable attackers.

We illustrate the concrete utility of the model by simulating two policy dilemmas: first the fairness of uniform security taxes, and then what happens in presence of a technological investment that changes the degree of interdependence for some airports but not for all. We demonstrate that several implicit transfers occur that result in cost externalities impacting smaller airports that are unable to take advantage of the economies of scale of the large regional hubs. (In Fig. 4, the policy maker is actually collecting a higher fee from small airport passengers that they would otherwise collect by themselves.) We also show that this channel of externalities can be inverted or magnified (under differing technology conditions) if some of the smaller airports collectively virtualize and centralize airport control tower operations into a through scheme resulting in a leap in block dependency also for airports that should be unaffected (see, e.g., Fig. 5).

Our general model of interdependent security investment and our simulation based on airport traffic data demonstrates that security investment shortfalls are heavily clustered with a substantial degree of separation between the larger integrated regional hubs and the smaller point to point airports. Hence a one‐size‐fits‐all policy may be far from optimal.
